# Validity and reliability of a home environment inventory for physical activity and media equipment

**DOI:** 10.1186/1479-5868-5-24

**Published:** 2008-04-29

**Authors:** John R Sirard, Melissa C Nelson, Mark A Pereira, Leslie A Lytle

**Affiliations:** 1 Division of Epidemiology and Community Health, School of Public Health, University of Minnesota, Minneapolis, MN, USA

## Abstract

**Background:**

Little is known about how the home environmental supports physical activity and screen media usage. The purpose of this study was to develop and test the reliability and validity of a self-report instrument to comprehensively reflect the availability and accessibility of physical activity and screen media equipment in the home environment.

**Methods:**

Ten families participated in the initial field testing to provide feedback for instrument development. Thirty one adult participants, each of whom had at least one child 10–17 years old, completed two Physical Activity and Media Inventory (PAMI) instruments. The first PAMI was completed simultaneously, but independently, with a research assistant to assess validity. A second PAMI was completed by the participant one week later to assess reliability.

**Results:**

The adult participants were mostly mothers/female guardians, mean age 38 ± 7.2 years, mostly Caucasian (52%), college educated (65%), living in single family homes (74%). Test-retest reliability was acceptable to strong for all summary variables (physical activity equipment, ICC = 0.76 to 0.99; media equipment, ICC = 0.72 to 0.96). For validation, reports from participants and research assistants were strongly correlated (physical activity, 0.67 – 0.98; media, 0.79 – 0.96). Compared to participants, research assistants reported a greater percentage of physical activity equipment as "in plain view and easy to get to" and a smaller percentage of items as "put away and difficult to get to".

**Conclusion:**

Our results indicate strong evidence for the reliability and validity of the variables calculated from the PAMI. This self report inventory may be useful in assessing the availability of physical activity and screen media equipment in the home environment and could be used in conjunction with other home assessment tools (food availability, parenting styles and feeding practices) to identify obesogenic home environments.

## Background

The use of ecological models to describe and understand physical activity and other health behaviors calls for new strategies for assessing and intervening upon the social and physical environments that affect those health behaviors [1]. While most attention has been paid to assessing activity options in the neighborhood environment [2-5], less attention has been focused on the home environment. The home environment, including areas both inside and outside (e.g., yard, garden) as well as items in the home that either facilitate or discourage activity, may have an important influence on physical activity patterning and warrants further study.

Valid and reliable methods of assessing environmental supports for physical activity and competing sedentary media pursuits within the home environment are needed. Assessments of both the activity and sedentary environmental supports are warranted since physical activity and sedentary behavior are unique and independent behaviors [6,7]. A person can be physically active but also engage in substantial amounts of sedentary activities like watching television or playing video games [7]. Particularly in children, there is good evidence that television time is related to the prevalence of childhood obesity [8,9].

To date, most physical activity inventories have shown weak (r = 0.04 to 0.19) [10] or no association [11] between physical activity and children's perception of their access to sporting and fitness equipment at home. In a study of college students, the quantity of home exercise equipment based on dichotomous (yes/no) response checklists was only associated with strength training activity in bivariate analyses (r = 0.18) [12]. In another study of college students, a similar checklist was also associated with strength exercise, not total physical activity, using multivariate analyses (p = 0.005) [13]. These low associations may suggest that equipment is not related to activity or may reflect methodological issues. A more comprehensive assessment of the home environment may help to better explain physical activity and sedentary behaviors and the specific activities being performed. Other recent work by Hume et al. [14] has sought to develop a similar checklist-based tool to assess the home environment for physical activity among Australian children. Evaluation suggested that test-retest reliability of this instrument was high for most items (Kappa = 0.85 to 1.00). However, the validity of the tool was not examined, and thus this tool may suffer similar shortcomings as those checklists previously described. While the checklist-based instruments assess the breadth of items (the number of unique items), a more comprehensive assessment would also measure magnitude (how many of each item are present), a characteristic of the home environment which may provide an important influence on behavior.

In addition, previous home environment assessment tools have focused on availability, not accessibility. Accessibility is related to ease of use and cueing of behavior. A toy or piece of sports equipment that is readily available and easy to access poses a smaller barrier to use than one that must be retrieved from some inconvenient space. Likewise, the presence of a television set in the bedroom or a basketball hoop in the yard may serve as a visual cue to make use of those pieces of equipment. Similarly, stimulus control (i.e. controlling stimuli that can encourage or discourage a behavior) is recognized as one of the important processes of change used in conjunction with the Transtheoretical Model of behavior change [15] and has been associated with change from contemplation to preparation stages [16]. An inventory that can help evaluate access may be an important contribution to measurement tools.

The purpose of this study was to develop and test the reliability and validity of a self-report survey instrument to comprehensively reflect the availability and accessibility of physical activity and media equipment in the home environment. It is our hope that such an instrument may help us better understand how the environment impacts the health and health behaviors of families.

## Methods

This research was conducted as part of the Identifying Determinants of Eating and Activity (IDEA) study for the purpose of designing and assessing the reliability and validity of an instrument to assess the home environment with regard to equipment related to physical activity and screen time. IDEA is an etiological study to evaluate potential determinants of the development of overweight and obesity in youth; it is being conducted in the metropolitan area of Minneapolis and St. Paul. IDEA assesses potential obesogenic factors in youth's personal, family, home, school, and neighborhood environments using a longitudinal cohort design (Lytle, in review).

### Physical Activity and Media Inventory (PAMI): instrument development

The PAMI was designed to be a self-report inventory of both the availability and accessibility of equipment and other resources that may support family members' participation in activity and sedentary behaviors. We were interested in creating an instrument that documented the physical environment, not individual behavior within the environment, therefore, we chose not to assess the frequency with which equipment inventoried was used.

The development of the PAMI began by examining and adapting existing inventory instruments to address the specific needs of our research objectives and by creating a draft inventory. This measurement tool went through multiple drafts where face validity, clarity of the questions and format, and feasibility of administration was reviewed by study investigators and with other researchers who study family health. When a satisfactory draft was complete, we asked a convenience sample of ten families, recruited through local park and recreation centers, to complete and comment on the inventory. All participants were able to complete the PAMI and feedback suggested that the PAMI would be feasible as a self-report instrument. Based on their feedback, we further refined the instrument to improve instruction clarity and form layout; this version was used to assess the reliability and validity of the PAMI instrument.

The PAMI version that was evaluated in this reliability/validity study included a list of 50 physical activity equipment items grouped by the following categories: sports equipment, fitness equipment, transportation equipment, foot wear, water sports, and outdoor/yard (see Additional File 1). There also were five media equipment items listed: television, video cassette recorder (VCR) and/or DVD, digital video recorder (DVR) and/or TiVo, video game system, computer (desktop or laptop). To obtain more information on media, six media-related questions were asked including:

1. Type of television programming available (No TV, No cable, Basic Cable, Cable + Premium channels, Satellite/Dish),

2. Number of channels received (No TV, <15, 15–30, 31–45, 46–60, >60),

3. Number of videos and DVDs in the home (0, 1–25, 26–50, 51–75, 76–100, >100),

4. Number of video and computer games in the home (0, 1–10, 11–20, 21–30, 31–40, 41–50, >50),

5. Type of internet service (No internet service, Dial-up modem, DSL Modem, Cable Modem, Don't know),

6. Size of the primary television in the home (diagonal screen size, in inches).

Opposite the list of items there was a list of 16 rooms (e.g., living room, kitchen, etc...) which also included Yard/Outdoor Space, Attic/Basement/Storage Area, Garage, and Automobile(s) where respondents indicated the location of the available activity or media equipment. There was space for three "other" rooms in case the home had a room not listed (e.g., sunroom) or more space was needed for a particular location (most common for the garage).

To determine validity, one parent/guardian from each participating family completed the PAMI while a trained research assistant simultaneously completed an inventory in the family's home. To determine reliability, the same parent/guardian completed a second inventory one week after the first administration. The second inventory was done without the presence of a research assistant. All data were collected during May-June, 2006.

### Subjects

Families were recruited to participate in this study through posted flyers and staffed information booths at four Minneapolis Park and Recreation Department buildings. Eligibility criteria included that each family had at least one child between the ages of 10 and 18 and an adult (parent/guardian) willing to complete the inventory and permit a home visit for validation purposes. Written informed consent was obtained prior to all data collection activities. The University of Minnesota Institutional Review Board approved this study.

### Procedures

At the first visit, one adult household member and a research staff member did a walk through of the house, independently completing the PAMI instrument. The respondents were asked to walk through each room and indicate which physical activity and media equipment items were present (using code numbers from the list) and to rate the accessibility of each item. Access response options were: put away and difficult to get to; put away and easy to get to; in plain view and difficult to get to; in plain view and easy to get to. The research assistant independently completed the criterion PAMI at the same time with as little communication with the participating family member as possible. If a participant began speaking, the research assistant would ask them not to speak. Due to the potentially intrusive nature of such an inventory, the research assistant only inventoried those items that were in plain view, without moving or looking under furniture or opening closets, unless the participant did so first.

At the end of the first visit, the research assistant left a blank PAMI instrument with the participant. The participant was asked to independently complete their second PAMI approximately one week later using the same approach as the first administration. A self-addressed stamped envelope was provided for mailing the completed form back to research staff. Reminder messages (telephone and email) were made to families one day prior to the day when the second inventory was to occur.

Demographics were collected by self report during the first PAMI administration and included the age, race, and education level for each member of the household and one question asking about their homeowner status (i.e., apartment, condominium, multi-family house, or single-family house).

### Data reduction

The PAMI data were reduced to the following primary variables, calculated separately for physical activity and media equipment: total number of items, the density of items in the home (total number of items divided by the total number of rooms/locations), the number of items located in bedrooms, the density of items in bedrooms, the number of televisions in the home, and the number of televisions in bedrooms. For comparability to a checklist type of instrument [12,13], which record only the presence or absence or an item and not the quantity, the number of unique items reported was separately summed for physical activity equipment. For example, if more than one bicycle was reported on the PAMI, bicycle was only counted once to most closely represent a checklist type instrument.

We also created and assessed two summary scores. First, we calculated separate summary scores that accounted for availability and accessibility of the physical activity equipment (Physical activity Availability and Accessibility Summary Score (PAASS)) and media equipment (Media Availability and Accessibility Summary Score (MAASS)). Each item was multiplied by the accessibility code with 1 = "put away and difficult to get to" and 4 = "in plain view and easy to get to". A higher score reflects a greater overall presence in the home (both availability and accessibility).

To provide more detail, we examined specific categories of items, including; sports equipment, fitness equipment, transportation equipment, water sports equipment, and outdoor/yard equipment). Within each of these categories, we calculated the total number of items and mean accessibility ratings for all items within each category.

To rank the overall quality of the home, an overall home environment score was also calculated as the ratio of the PAASS to the MAASS (referred to as the Activity:Media Ratio Score). A higher overall Activity:Media Ratio Score would reflect a home more conducive for being physically active and less sedentary.

### Statistical analysis

SAS version 9.1 (Cary, NC) was used for all data analyses. A p ≤ 0.05 significance level was used as a guide for identifying significant relationships. Test-retest reliability of the continuous variables from the PAMI (i.e., number and density of items, number and density of items in bedrooms, checklist quantity, summary scores, number of physical activity items within categories, and overall home environment score) was assessed by intraclass correlation coefficient (ICC, 95% confidence intervals). Test-retest of the accessibility ratings (overall for the home and by physical activity item categories) was assessed by Mantel-Hanzel chi square analyses. The reliability of the additional media-related questions was assessed by percent agreement (Kappa coefficients, 95% Confidence Intervals).

Validity was evaluated by examining agreement between data from the participants and research assistants using Pearson correlation coefficients for continuous variables. Mean differences in continuous variables between the participant and the research assistant were identified with two-tailed independent t-tests. Comparison of the accessibility ratings between the participant and research assistant was calculated by Mantel-Hanzel chi square analyses.

## Results

A total of 31 families agreed to participate in the study and data from all families are included in these analyses. All participating families completed the PAMI at least once for validation purposes, and over 77% also completed the PAMI a week later to assess reliability. The PAMI took, on average, 40 minutes to complete but was dependent on the size of the home and the number of items present.

Table 1 provides the demographic characteristics of the participating families for the validation (N = 31) and reliability (N = 24) samples. Dependent t-tests and chi square analyses revealed no demographic differences in the families included in the validity and reliability samples (p = 0.26–0.94). The majority of parents/guardians were female, Caucasian and had at least a college education. On average, there were approximately four people per home, most with two adults and two children living in single-family homes.

**Table 1:** Subject characteristics for the validity and reliability samples

	**Validity Sample**	**Reliability Sample**
**Variable**	**N = 31**	**N = 24**
**Age, mean ± SD**	38 ± 7.2	37 ± 6.6
**Race/Ethnicity, n (%)**		
Caucasian	16 (52%)	12 (50%)
African American	6 (19%)	6 (25%)
Mexican-American, Puerto Rican, Latin American	2 (6%)	1 (4.2%)
Native American	2 (6%)	1 (4.2%)
Asian American	2 (6%)	1 (4.2%)
Other/Unknown	3 (10%)	3 (13%)
**Highest Education, n (%)**		
Did not graduate high school	2 (6%)	1 (4%)
High school/Trade school/Some college	7 (22%)	5 (21%)
> College graduate	20 (65%)	14 (58%)
Not answered	2 (6%)	4 (17%)
**Number of people in home, mean (SD)**	3.9 (0.9)	3.8 (1.1%)
**Number of adults in home (≥ 18 yrs), n (%)**		
1 Adult	10 (32%)	10 (42%)
2 Adults	20 (65%)	13 (54%)
3 or More Adults	1 (3%)	1 (4%)
**Number of minors in home (< 18 yrs), n (%)**		
1 Child	5 (16%)	4 (17)
2 Children	17 (55%)	13 (54)
3 Children	6 (19%)	5 (21)
4 Children	3 (10%)	2 (8)
**Type of home, n (%)**		
Apartment	2 (6%)	2 (8%)
Multi-family house	5 (16%)	5 (21%)
Single-family house	23 (74%)	15 (63%)
Not answered	1 (3%)	2 (8%)

### Reliability

Table 2 contains the results of the reliability analyses. Test-retest for the physical activity and media equipment variables was good to excellent (ICC = 0.71 to 0.96). Use of a checklist approach results in high reliability for the physical activity equipment (ICC = 0.93). The proportion of physical activity and media equipment items in each accessibility category was similar between the first and second administration (Mantel-Hanzel chi square tests, p = 0.50 and 0.30, respectively). The calculated PAASS and MAASS (number of items * accessibility) revealed high reliability between the first and second administration of the PAMI (ICC= 0.87 for physical activity items and ICC = 0.93 for media equipment).

**Table 2:** One-week test-retest reliability of PAMI variables; mean ± SD with Intraclass Correlation Coefficient (ICC) and 95% Confidence Interval (CI) or percent with Mantel-Hanzel Chi Square

**Variable**	**Time 1**	**Time 2**	**ICC (95% CI)**
Total number of rooms	11.9 ± 3.10	12.5 ± 3.22	0.86 (0.70 – 0.94)
Physical Activity Equipment			
# of Items	65.7 ± 56.52	64.8 ± 58.06	0.94 (0.87 – 0.98)
Household Density	5.1 ± 4.16	5.0 ± 4.53	0.84 (0.86 – 0.97)
Ave # of Items/Bedroom	3.3 ± 3.10	3.5 ± 3.30	0.76 (0.51 – 0.89)
Checklist Quantity	16.4 ± 7.85	17.5 ± 7.63	0.93 (0.84 – 0.97)
Accessibility			
Away and difficult to get to	8.7	4.4	M-H χ^2 ^= 0.46, p = 0.50
Away and easy to get to	17.8	21.5	
In view and difficult to get to	2.9	2.3	
In view and easy to get to	70.3	71.9	
PAASS	207.4 ± 166.74	216.8 ± 190.12	0.87 (0.74 – 0.94)
			
Media Equipment			
# of Items	8.4 ± 5.02	8.4 ± 4.93	0.96 (0.91 – 0.98)
Household Density	0.73 ± 0.38	0.73 ± 0.43	0.89 (0.76 – 0.95)
Ave # of Items/Bedroom	2.4 ± 0.78	2.1 ± 0.68	0.72 (0.44 – 0.87)
# of Televisions	2.5 ± 1.20	2.7 ± 1.27	0.89 (0.76 – 0.95)
# of Televisions in Bedrooms	1.17 ± 0.94	1.27 ± 1.03	0.93 (0.82 – 0.97)
Accessibility			
Away and difficult to get to	1.4	2.6	M-H χ^2 ^= 1.05, p = 0.30
Away and easy to get to	10.3	12.7	
In view and difficult to get to	0.7	1.3	
In view and easy to get to	87.6	83.4	
MAASS	32.0 ± 25.5	31.2 ± 18.84	0.93 (0.84 – 0.97)

PAASS, Physical activity Availability and Accessibility Summary Score

MAASS, Media Availability and Accessibility Summary Score

The test-retest reliability for the number of items within all physical activity equipment categories was high (ICC = 0.87 to 0.99) (Table 3). Reliability of accessibility ratings was less consistent across the categories. However, low cell sizes (n ≤ 5) for most of these analyses indicate that these results should be interpreted with some caution.

**Table 3:** Reliability statistics for physical activity equipment separated by categories; Mean (SD) for participant data at Time 1 and Time 2, Intraclass Correlation Coefficient (ICC) and 95% Confidence Intervals (95% CI) or Mantel-Hanzel Chi Square test

	**Time 1**		**Time 2**		**T1 vs T2**
		
**Item Category**	**n**	**mean (SD)**	**n**	**mean (SD)**	**ICC (95% CI)**
**Sports Equipment**					
# of Items	23	34.6 (36.50)	23	33.5 (38.6)	0.96 (0.90 – 0.98)
Accessibility					
Away and difficult to get to		14.2		8.6	M-H χ^2 ^= 64.2, p < 0.01
Away and easy to get to		25.2		27.7	
In view and difficult to get to		3.5		5.1	
In view and easy to get to		57.1		58.6	

**Fitness Equipment**					
# of items	21	7.0 (4.69)	21	7.3 (5.93)	0.95 (0.88 – 0.98)
Accessibility					
Away and difficult to get to		6.6		2.2	M-H χ^2 ^= 5.63, p = 0.02
Away and easy to get to		28.6		31.1	
In view and difficult to get to		3.3		2.2	
In view and easy to get to		61.5		64.4	

**Transportation Equipment**					
# of items	23	7.3 (4.58)	22	7.8 (5.42)	0.88 (0.74 – 0.94)
Accessibility					
Away and difficult to get to		2.9		0.0	M-H χ^2 ^= 20.4, p < 0.01
Away and easy to get to		25.7		28.2	
In view and difficult to get to		4.3		2.8	
In view and easy to get to		67.1		69.0	

**Water Sports Equipment**					
# of items	15	5.5 (6.09)	15	5.8 (9.01)	0.99 (0.97 – 1.00)
Accessibility					
Away and difficult to get to		12.5		7.7	M-H χ^2 ^= 0.02, p = 0.87
Away and easy to get to		45.8		26.9	
In view and difficult to get to		4.2		3.9	
In view and easy to get to		37.5		61.6	

**Outdoor/Yard Equipment**					
# of items	20	12.5 (9.90)	20	13.5 (9.46)	0.87 (0.70 – 0.95)
Accessibility					
Away and difficult to get to		13.7		6.1	M-H χ^2 ^= 8.3, p < 0.01
Away and easy to get to		9.2		20.6	
In view and difficult to get to		4.6		2.3	
In view and easy to get to		72.5		71.0	

The test-retest reliability for the additional media questions ranged from K = 0.42 (0.10 to 0.73) for the number of video and computer games to K = 1.00 for the type of television and for the type of internet service. The Kappa statistics for the number of videos/DVDs (K = 0.60, 0.36 to 0.84) and number of television channels (K = 0.87, 0.71 to 1.00) were both acceptable. The Kappa statistic for television size (K = 0.71, 0.35 to 1.00) is based on categorizing the responses into small (<25"), medium (25–39"), and large (> = 40").

### Validity

Table 4 contains the results of the validation analyses. Associations between the participant and research assistants were moderate to strong across all variables (r = 0.67 to 0.98) and there were no significant differences between the mean values recorded by participants and research assistants (t-test p-values 0.20 to 0.72). For the accessibility ratings, the participants, compared to the research assistants, recorded a greater percentage of physical activity items as "put away and difficult to get to" and a smaller percentage of items as "in plain view and easy to get to" (p < 0.001). No differences were observed for the accessibility ratings of the media items (p = 0.23). The PAASS was significantly greater for the research assistants compared to the participants (p = 0.03). There was no mean difference in the MAASS (p = 0.30).

**Table 4:** Validity of PAMI variables; Mean ± SD; (N = 31) mean ± SD with Pearson Correlation Coefficient and p-value for independent t-test or percent with Mantel-Hanzel Chi Square

**Variable**	**Participant Time 1**	**Research Assistant**	**Correlation (Pearson r)**	**t-test p-value**
Total number of rooms	12.4 ± 2.91	12.0 ± 2.42	0.72*	0.34
Physical Activity Equipment				
# of items	65.2 ± 54.12	74.8 ± 64.77	0.98*	0.53
Household Density	5.0 ± 3.95	6.2 ± 5.54	0.93*	0.33
Ave # of Items/Bedroom	2.1 ± 2.68	2.8 ± 4.04	0.67*	0.25
Checklist Quantity	16.8 ± 7.46	17.2 ± 7.27	0.94*	0.36
Accessibility				
Away and difficult to get to	9.5	2.5	--	M-H χ^2 ^= 16.5,
Away and easy to get to	20.1	22	--	p < 0.001
In view and difficult to get to	5.3	2	--	
In view and easy to get to	65	73.6	--	
PAASS	209.9+162.24	261.2+231.3	0.93*	0.03
			
Media Equipment				
# of items	8.5 ± 4.49	8.8 ± 4.47	0.93*	0.20
Household Density	0.71 ± 0.34	0.75 ± 0.34	0.79*	0.33
Ave # of Items/Bedroom	1.7 ± 1.20	1.7 ± 1.20	0.94*	0.49
# Televisions in Home	2.5+1.23	2.9+1.44	0.87*	0.25
# Televisions in Bedrooms	1.16+1.04	1.26+1.09	0.96*	0.72
Accessibility				
Away and difficult to get to	1.0	1.3	--	M-H χ^2 ^= 1.4,
Away and easy to get to	9.3	6.5	--	p = 0.23
In view and difficult to get to	2.5	0.0	--	
In view and easy to get to	87.3	92.2	--	
MAASS	31.9+25.53	34.0+17.27	0.88*	0.30

PAASS, Physical activity Availability and Accessibility Summary Score

MAASS, Media Availability and Accessibility Summary Score

* p < = 0.05

Table 5 contains the results comparing the participant (at time 1) and the research assistant separated by the physical activity equipment categories. Across categories, associations were generally high (r = 0.80 to 0.98) except for the "transportation equipment" category (r = 0.68). Research assistants recorded more sports equipment (t-test p < 0.01), compared to the participants. Comparisons of the accessibility ratings indicate that accessibility for fitness, transportation, and water sports equipment were rated similarly between participants and research assistants (p = 0.23 to 0.73). However, research assistants recorded a greater proportion of sports and outdoor/yard equipment items as "in view and easy to get to" and a smaller proportion of items as "put away and difficult to get to".

**Table 5:** Validity statistics for physical activity equipment separated by categories; Mean (SD) for participant at Time 1 and Research Assistants with Pearson correlation coefficients, t-test p-value or Mantel-Hanzel Chi Square test

	**Time 1**		**RA**		**T1 vs RA**	
		
**Item Category**	**n**	**mean (SD)**	**n**	**mean (SD)**	**Correlation**	**t-test p-value**
**Sports Equipment**						
# of Items	31	33.5 (34.20)	31	42.9 (45.53)	0.98	< 0.01
Accessibility						
Away and difficult to get to		10.4		2.4	M-H χ^2 ^= 64.2, p < 0.01	
Away and easy to get to		24.5		28.0		
In view and difficult to get to		3.7		0.8		
In view and easy to get to		61.4		68.8		

**Fitness Equipment**						
# of items	28	6.4 (4.31)	28	6.4 (4.78)	0.82	0.99
Accessibility						
Away and difficult to get to		5.9		4.8	M-H χ^2 ^= 0.11, p = 0.73	
Away and easy to get to		25.5		26.7		
In view and difficult to get to		3.9		0.0		
In view and easy to get to		64.7		68.6		

**Transportation Equipment**						
# of items	28	7.7 (5.44)	31	7.8 (4.71)	0.68	0.90
Accessibility						
Away and difficult to get to		4.3		2.2	M-H χ^2 ^= 1.41, p = 0.23	
Away and easy to get to		16.1		14.3		
In view and difficult to get to		10.8		5.5		
In view and easy to get to		68.8		78.0		

**Water Sports Equipment**						
# of items	16	4.7 (5.75)	17	4.6 (5.58)	0.92	0.75
Accessibility						
Away and difficult to get to		12.0		0.0	M-H χ^2 ^= 0.29, p = 0.59	
Away and easy to get to		44.0		52.0		
In view and difficult to get to		4.0		8.0		
In view and easy to get to		40.0		40.0		

**Outdoor/Yard Equipment**						
# of items	28	12.8 (10.48)	28	14.4 (9.45)	0.80	0.17
Accessibility						
Away and difficult to get to		14.3		2.8	M-H χ^2 ^= 12.63, p < 0.01	
Away and easy to get to		11.2		10.7		
In view and difficult to get to		6.2		3.4		
In view and easy to get to		68.3		83.1		

Reliability and validity were also assessed for the overall home environment Activity:Media Ratio Score (the ratio of the PAASS to MAASS). At Time 1 and 2 the Activity:Media Ratio Score was 8.3 ± 7.98 and 8.3 ± 7.28, respectively (ICC = 0.91 (0.81 to 0.96)). There was a strong association between the participant- and research assistant-derived home environment scores (r = 0.94, P < 0.01) and no significant difference in scores (participant; 8.2 ± 7.40, research assistant; 9.5 ± 10.44, p = 0.09).

## Discussion

This study assessed the test-retest reliability and validity of a self-administered, physical activity and media equipment inventory instrument for the home environment. Reliability ICCs for all of the physical activity and media equipment variables were moderate to high. This was true for all of the continuous variables, including the checklist quantity and the physical activity and media summary scores. When broken down by categories of physical activity items, reliability of the number of items remained consistently high across categories. The accessibility ratings were similar between the two administrations of the PAMI for fitness, transportation, and water sports equipment. In contrast, the accessibility ratings for sports and outdoor/yard equipment were different with fewer items considered "put away and difficult to get to" at the second administration, compared to the first. The reason for this difference may be a real change in where items in the home were placed or in the participants' perception about the accessibility of the items.

Validity, as assessed by comparing the participant and research assistant data, was consistently high for all calculated variables for both physical activity and media equipment. The PAASS summary score was higher for the research assistants compared to the participants and likely reflects the research assistants recording more items as "in plain view and easy to get to" compared to the participants. When broken down by categories of physical activity equipment, validity coefficients for the number of items in each category were still generally high. The distribution of accessibility ratings for the media equipment were similar between participants and research assistants with most items being "in plain view and easy to get to". Research assistants, however, recorded a smaller percentage of physical activity items as "put away and difficult to get to" and a greater percentage of items as "in plain view and easy to get to" as compared with participants. The difference between participant and research assistant ratings of accessibility across all physical activity items seems to be driven by a difference in ratings for the sports and yard/outdoor equipment.

There may have been a discrepancy between what participants and research assistants considered "put away". While the participant may consider items placed on the floor by the kitchen door as, "put away" (if it was where those items are typically kept), the research assistants would have coded such items as, "in plain view". The term "put away" did not necessitate that the items be in a box, cabinet, drawer, or other closed container. In addition, this discrepancy may have occurred due to the protocol followed by the research assistants which allowed them to only record items that they could see, without opening doors, closets and looking under furniture (e.g., beds), unless the participant first did so. Our intention with implementing this protocol was to protect participant privacy. Further modification and testing of accessibility options is warranted given these findings.

This was a small pilot study with several limitations. The majority of respondents were Caucasian, college educated and lived in single family homes. Additional validation studies are warranted in larger and more representative samples to determine the PAMI's psychometric properties in minority and lower income populations and those who live in apartments or condominiums. Participants were recruited from local parks and recreation departments which may have biased our sample by including relatively active families with relatively large amounts of physical activity equipment. Several families, however, had very few physical activity items, evidenced by the large standard deviations for most of the physical activity equipment variables. While the list of physical activity items was fairly comprehensive, some items may be more relevant in certain geographical areas (i.e., snow skis in Minnesota). Therefore, the list of items may need to be modified based on the population being studied. Only screen based media items were included on the PAMI. A more comprehensive assessment of media sources in the home would need to include magazines, radios, portable music players, cell phones, and possibly other sources. During the validation visits, participants walked from room to room to complete the PAMI, as the instructions indicate. Whether this happened during the second administration of the PAMI, when the participants were by themselves, is not known. The PAMI is also not designed to assess how frequently or who in the home, is using the equipment. However, in an ecological model, characterizing the environment is an important end in and of itself as the environment is important in influencing behavioral choices. Lastly, data were collected in the spring and seasonality should be considered when interpreting the location and accessibility data since some items (e.g., snow skis) may be moved and made very accessible during the winter but stored away at other times of the year.

At present, it is not known if the PAMI is useful in characterizing homes along dimensions related to important health outcomes such as obesogeneity, levels of physical activity or sedentary behaviors. We also do not yet know if the PAMI is a useful instrument for showing change overtime as might be useful in intervention research that attempts to improve the healthfulness of the home environment. Future research will be needed to examine both the discriminant and predictive validity of the PAMI.

In spite of these limitations, the PAMI is a potentially important addition to the field. The PAMI provides a more objective assessment of the presence of physical activity and media equipment items as compared to other research examining children's perception of adequate equipment [10,11]. The PAMI instrument also expands on previous dichotomous checklist instruments [12,13] by measuring the breadth (the number of unique items), magnitude (how many of each type of item) and ratings of accessibility of the items.

### Lessons learned

In the PAMI instrument tested here (see Additional File 1), several types of foot wear were included as a category in the list of physical activity equipment (e.g., running shoes, hiking shoes, walking shoes, etc...). There was considerable disagreement and confusion on identifying these particular types of shoes and, for many younger children and some adults, one pair of athletic shoes may serve multiple roles (walking, running, hiking). Also, there were so many pairs of shoes per household (range 1 to 59) that the discriminative ability of the number/accessibility of shoes was quite dubious. Therefore, types of shoes were not included in the analyses for this paper. Briefly, the ICC for "shoes" was 0.82 (0.62 – 0.92) with a low correlation between the participant and the research assistant (r = 0.22). The participants reported slightly fewer pairs of shoes (14.7 ± 11.77), on average, compared to the research assistants (18.3 ± 12.25) (p = 0.07). For the main IDEA study, shoes were not included in the physical activity item list.

In addition, based upon feedback from participants and research assistants, the PAMI equipment code numbers (included on a separate page of the tool) were sometimes difficult for the participants to find and record. Therefore, for the main IDEA study, the PAMI has been expanded so that each room has its own page containing the list of items where the participant indicates how many of each item are present in that room and the associated accessibility. We believe the re-formatted version of the PAMI will be easier for participants to complete, although this newer version was not tested in this pilot study.

## Conclusion

The PAMI may be a useful tool for describing the physical home environment related to opportunities for physical activity, sedentary behaviors and possibly the obesogeneity of the home environment. The main IDEA study will assess the associations between PAMI variables, physical activity and sedentary behaviors, and weight status of both the adult and youth participating in the study. In addition, the longitudinal design of the IDEA study will allow us to look at physical activity and media equipment in the home environment as potential determinants of physical activity, sedentary behavior, and as factors related to the obesogeneity of home environments. In conclusion, the results from this study indicate that the variables calculated for this study (number of items, density, checklist quantity, accessibility, summary scores) provide reliable estimates for describing the home environment related to the presence of physical activity and screen media equipment. The validity of the accessibility ratings for the physical activity items was less consistent and requires further investigation.

## Authors' contributions

JS and LL conceived the study. All authors (JS, MN, MP, LL) contributed significantly to the development of the inventory instrument and the study design, manuscript development and revision. All authors read and approved the final manuscript.

## Supplementary Material

Additional file 1 The Physical Activity and Media Inventory (PAMI) instrument. The full PAMI instrument used for this study. Click here for file
