# Supplementary material for: Validity and reliability of a home environment inventory for physical activity and media equipment
Source: Int J Behav Nutr Phys Act. 2008 Apr 29;5:24. doi: 10.1186/1479-5868-5-24 (PMC2386867; doi:10.1186/1479-5868-5-24)
Supplement: Additional file 1 — The Physical Activity and Media Inventory (PAMI) instrument. The full PAMI instrument used for this study. [file 1479-5868-5-24-S1.doc]

ID:_____

***Physical Activity and Media Inventory***

We are interested in learning what types of physical activity and media equipment you have and where you keep these items.

If you have any questions about this survey or the study, please see the contact information at the back of this booklet. Thank you for helping us with this study!

**Please enter today’s date:** _______________

**Instructions**

1. Please walk through each room (yard, garage and automobiles, if present) and use the numbered list on the next page to indicate which items are in the room by writing the corresponding numbers in the top row of boxes (**see example below**). Write one item number per box.
2. Use the following list to indicate how accessible the item is by writing the letter in the bottom row of boxes
   1. Put away and difficult to get to (e.g., stored snow boots)
   2. Put away and easy to get to (e.g., VCR behind a cabinet door)
   3. In plain view and difficult to get to (e.g., snow skis stored in garage rafters)
   4. In plain view and easy to get to (e.g., skateboard on floor in entryway)

**Important Notes**

1. Please take the time to walk through your home rather than sitting in one place to complete this inventory. Walking through each room will help your memory.
2. If there is more than one of the same item in a room (two pairs of running shoes in the entry way), write the code number in the top left of the box and how many of the item in the lower right of the box (**see example below**).
3. If there are not enough boxes for all of the items in the room, use one of the “Other” rows and write in the name of the room.
4. If the room does not apply to your home, write “NA” in the first box for that room.
5. If there is nothing from the list in the room, write “0” in the “Item #” row.

| ***Example*** | | | | | | | | | |  | | | | | | | | | | |
| --- | --- | --- | --- | --- | --- | --- | --- | --- | --- | --- | --- | --- | --- | --- | --- | --- | --- | --- | --- | --- |
| Item # | ***27*** | ***29***  ***2*** | ***46*** | ***11*** |  |  |  |  |  |  |  |  |  |  |  |  |  |  |  |  |
| Accessibility | ***D*** | ***D*** | ***D*** | ***B*** |  |  |  |  |  |  |  |  |  |  |  |  |  |  |  |  |

| **Physical Activity and Media Equipment Item Numbers** | |
| --- | --- |
| **# Sports Equipment**  1 Backstop (baseball, soccer, hockey)  2 Balls (soccer, football, basketball, baseball)  3. Baseball bat / t-ball equipment  4 Baseball/softball glove  5 Basketball hoop  6 Frisbee  7 Golf clubs  8 Helmet / Protective gear  9 Ping pong table  10 Racquet (tennis, badminton)  11 Skates (roller / in-line / ice)  12 Skis (downhill, cross-country)  13 Snowboard  14 Snow shoes (pairs)  **# Fitness Equipment**  15 Aerobic workout videos  16 Exercise / yoga mat  17 Jump rope  18 Stationary exercise equipment  (treadmill, bike, step/slide aerobic)  19 Trampoline  20 Weight lifting / resistance training equipment  **# Transportation Equipment**  21 Bicycle, tricycle  22 Bicycle trailer  23 Jogging Stroller  24 Scooter  25 Skateboard  **# Athletic Footwear**  26 Cleats / sports shoes (pairs)  27 Comfortable walking shoes (pairs)  28 Hiking boots/shoes (pairs)  29 Running shoes (pairs)  30 Snow boots (pairs) | **# Water Sports**  31 Canoe / Kayak / Sail boat  32 Pool toys  33 Surf / boogie board  34 Water skis  35 Wind surf / sail board  **# Outdoor / Yard Equipment**  36 Gardening tools  37 Lawn mower - push  38 Lawn mower- riding  39 Leaf blower  40 Net (volleyball, badminton)  41 Play structure (swings, slide, climbing)  42 Pool (in ground or above)  43 Rake  44 Sandbox  45 Snow blower  46 Snow shovel  47 Snow sled  48 Trampoline  49 Yard game (croquet, horseshoes)  50 Yard tools (clippers, wheelbarrow)  **# Working Media Equipment**  51 Television  52 VCR / DVD Player  53 Digital Video Recorder / TiVO  54 Video game system (Portable/Stationary) (X-Box, Ninendo, GameBoy)  55 Computer (laptop, desktop) |

**Accessibility List**

| ***Example*** | | | | |
| --- | --- | --- | --- | --- |
| Item # | ***27*** | ***29*** | ***46*** | ***11*** |
| Accessibility | ***D*** | ***D*** | ***D*** | ***B*** |

***2***

- 1. Put away and difficult to get to (e.g., stored snow boots)
  2. Put away and easy to get to (e.g., VCR behind a cabinet door)
  3. In plain view and difficult to get to (e.g., snow skis stored in garage rafters)
  4. In plain view and easy to get to (e.g., skateboard on floor in entryway)

| **Entryway / Foyer / Mudroom** | | | | | | | | | | **Bedroom 1 (Adult ⬜ or Child ⬜) check one** | | | | | | | | | | |
| --- | --- | --- | --- | --- | --- | --- | --- | --- | --- | --- | --- | --- | --- | --- | --- | --- | --- | --- | --- | --- |
| Item # |  |  |  |  |  |  |  |  |  |  |  |  |  |  |  |  |  |  |  |  |
| Accessibility |  |  |  |  |  |  |  |  |  |  |  |  |  |  |  |  |  |  |  |  |
| **Porches / Decks (all)** | | | | | | | | | | **Bedroom 2 (Adult ⬜ or Child ⬜) check one** | | | | | | | | | | |
| Item # |  |  |  |  |  |  |  |  |  |  |  |  |  |  |  |  |  |  |  |  |
| Accessibility |  |  |  |  |  |  |  |  |  |  |  |  |  |  |  |  |  |  |  |  |
| **Living Room** | | | | | | | | | | **Bedroom 3 (Adult ⬜ or Child ⬜) check one** | | | | | | | | | | |
| Item # |  |  |  |  |  |  |  |  |  |  |  |  |  |  |  |  |  |  |  |  |
| Accessibility |  |  |  |  |  |  |  |  |  |  |  |  |  |  |  |  |  |  |  |  |
| **Dining Room** | | | | | | | | | | **Bedroom 4 (Adult ⬜ or Child ⬜) check one** | | | | | | | | | | |
| Item # |  |  |  |  |  |  |  |  |  |  |  |  |  |  |  |  |  |  |  |  |
| Accessibility |  |  |  |  |  |  |  |  |  |  |  |  |  |  |  |  |  |  |  |  |
| **Den / Office** | | | | | | | | | | **Attic / Basement / Storage Area** | | | | | | | | | | |
| Item # |  |  |  |  |  |  |  |  |  |  |  |  |  |  |  |  |  |  |  |  |
| Accessibility |  |  |  |  |  |  |  |  |  |  |  |  |  |  |  |  |  |  |  |  |
| **Kitchen** | | | | | | | | | | **Garage 1** | | | | | | | | | | |
| Item # |  |  |  |  |  |  |  |  |  |  |  |  |  |  |  |  |  |  |  |  |
| Accessibility |  |  |  |  |  |  |  |  |  |  |  |  |  |  |  |  |  |  |  |  |
| **Family Room** | | | | | | | | | | **Garage 2** | | | | | | | | | | |
| Item # |  |  |  |  |  |  |  |  |  |  |  |  |  |  |  |  |  |  |  |  |
| Accessibility |  |  |  |  |  |  |  |  |  |  |  |  |  |  |  |  |  |  |  |  |
| **Bathrooms (all)** | | | | | | | | | | **Automobile(s)** | | | | | | | | | | |
| Item # |  |  |  |  |  |  |  |  |  |  |  |  |  |  |  |  |  |  |  |  |
| Accessibility |  |  |  |  |  |  |  |  |  |  |  |  |  |  |  |  |  |  |  |  |
| **Other (please specify) ____________________** | | | | | | | | | | **Yard / Outdoor Space** | | | | | | | | | | |
| Item # |  |  |  |  |  |  |  |  |  |  |  |  |  |  |  |  |  |  |  |  |
| Accessibility |  |  |  |  |  |  |  |  |  |  |  |  |  |  |  |  |  |  |  |  |
| **Other (please specify) ____________________** | | | | | | | | | | **Other (please specify) __________________** | | | | | | | | | | |
| Item # |  |  |  |  |  |  |  |  |  |  |  |  |  |  |  |  |  |  |  |  |
| Accessibility |  |  |  |  |  |  |  |  |  |  |  |  |  |  |  |  |  |  |  |  |

***Additional Media Questions***

**Instructions: Please circle only one answer for each.**

1. How many channels do you receive on your television (the primary television in the home)?

No TV in the home < 15 15-30 31-45 46-60 >60

2. What best describes your television service for the primary television in the home?

1. No TV in the home 2. No cable 3. Basic cable 4. Cable + premium channels 5. Satellite/Dish

3. How many videos and/or DVDs do you currently have in your home?
 Include items that are owned, rented and borrowed.

0 1-25 26-50 51-75 76-100 >100

4. How many video games and computer games are in your home?
 Include items that are owned, rented and borrowed.

0 1-10 11-20 21-30 31-40 41-50 >50

5. What best describes your type of internet service?

1. No internet access 2. Dial-up modem 3. DSL Model 4. Cable Modem 5. Don’t Know

6. What is the size of the primary television in the home? Please measure your TV screen diagonally if you are not sure.

_________ inches (***Diagonal*** *screen size*)

***About You and Your Family***

**Instructions**: In the following table, please list all of the people in your home, their age, race/ethnicity and education level. Instead of using names, identify people by their relation to you (husband, daughter, son, etc…). Use the first line of the table for yourself. Use the race/ethnicity and education code numbers provided below.

| **Race/Ethnicity code numbers** |  | **Education code numbers** |
| --- | --- | --- |
| 1. African American |  | 1. Too young for elementary school |
| 2. Asian / Pacific Islander |  | 2. Currently enrolled in elementary, middle, or high school |
| 3. Caucasian |  | 3. Did not finish high school |
| 4. Hispanic / Latino |  | 4. Finished high school (or got a GED) |
| 5. Native American |  | 5. Went to vocational school (Computer/electrician/mechanic) |
| 6. Multi-racial |  | 6. Some college (but did not graduate) |
| 7. Other |  | 7. Graduated from college or a university |
|  |  | 8. Some professional training beyond a 4-year college degree |
|  |  | 9. Don’t know |

| **Example** | | | | |
| --- | --- | --- | --- | --- |
|  | **Person** | **Age** | **Race/Ethnicity** | **Education** |
| 1 | ***Son*** | ***12*** | ***3*** | ***2*** |

|  | **Person** | **Age** | **Race/Ethnicity** | **Education** |
| --- | --- | --- | --- | --- |
| 1 | (you) |  |  |  |
| 2 |  |  |  |  |
| 3 |  |  |  |  |
| 4 |  |  |  |  |
| 5 |  |  |  |  |
| 6 |  |  |  |  |
| 7 |  |  |  |  |
| 8 |  |  |  |  |

How many dogs are in your home? (**please circle one**)

0 1 2 3 or more

What best describes your home? (**please circle one**)

1. Apartment

2. Condominium

3. Multi-family house (duplex)

4. Single family house

***Thank You !***
